# Dynamic Evaluation Indices in Spatial Learning and Memory of Rat Vascular Dementia in the Morris Water Maze

**DOI:** 10.1038/s41598-019-43738-x

**Published:** 2019-05-10

**Authors:** Ze Yuan, Hongying Zhou, Ni Zhou, Dong Dong, Yuyang Chu, Junxian Shen, Yunfeng Han, Xiang-Ping Chu, Kunjie Zhu

**Affiliations:** 1 School of Mental Health, Qiqihar Medical University, Qiqihar, Heilongjiang 161006 China; 2 Feinberg School of Medicine, Northwestern University, Chicago, Illinois 60611 USA; 3 College of Aerospace and Civil Engineering, Harbin Engineering University, Harbin, Heilongjiang 150001 China; 4 School of Public Health, Qiqihar Medical University, Qiqihar, Heilongjiang 161006 China; 5 Department of Biomedical Sciences, School of Medicine, University of Missouri-Kansas City, Missouri, 64108 USA; 6 School of Basic Medicine, Qiqihar Medical University, Qiqihar, Heilongjiang 161006 China

**Keywords:** Neuroscience, Spatial memory

## Abstract

The Morris water maze (MWM) is widely used to evaluate rodent spatial learning and memory. However, current evaluation measures are not comprehensive because there is a wide distribution in the measured response. Utilizing the graph cognition hypothesis, we proposed four new deviation indices to evaluate cognitive function in the MWM that compared the optimal swim track to the actual track taken. These include the sum of the lateral deviation vectors, the sum of the offset angles, the sum of the correction vectors, and the sum of the lateral deviation vectors to the initial optimal route. We compared the four new deviation indices to the classically used escape latency measures in a vascular dementia model and demonstrated a higher consistency in the normal distribution between the vascular dementia group and the control rats. Further, the new measures displayed higher sensitivity and specificity compared to what escape latency displayed in the Monte Carlo simulation. From the receiver operating characteristic curve, the diagnostic values of the new deviation indices are higher than those of escape latency. Therefore, including these new evaluation indices in MWM experiments provided a more effective analysis of cognitive function compared to using escape latency.

## Introduction

The Morris water maze (MWM) evaluates the spatial learning and memory ability of rodents^1–3^, and it is widely used to examine hippocampal-dependent memory^4^. Many of the metrics currently used in the MWM are based on simple summaries of the rodent’s path, such as path-length, the percent of time spent away from walls, escape latency, angles of approach to the platform, and quadrant time, etc.^5,6^. These measures are convenient to observe and record, but they rarely examine the dynamic behavior of the animal while searching for the platform^7^, missing key parameters that could provide valuable insights into learning and memory. For example, escape latency, one of the most common parameters, was widely used in the MWM^7,8^. Reductions in escape latency suggest that the experimental subjects focused on searching strategies, but in fact, reduced escape latencies may also reflect the adoption of non-spatial strategies^9^. Rodents that suffer from vascular dementia swim along the wall randomly searching for escape routes until they happen to swim away from the wall and find the hidden platform. The faster they swim, the more likely they are to encounter the platform randomly. Therefore, the low escape latency in a single training does not reflect the ability of the subject to use spatial cues to find the platform.

Several recent studies suggested that using escape latency to evaluate learning and memory is not comprehensive^7,8^. Therefore, total distance, target quadrant activity time, searching strategies and other indices are also used as common reference indices to comprehensively evaluate the learning and memory abilities of the experimental subjects^9–11^. These parameters compensate for deficiencies in escape latency measures, but there is a clear separation between these indices and escape latency. For example, if the subject used less time (escape latency) to find the platform, the target quadrant time spent by the subject cannot support this data. In order to resolve such contradictions, Maei *et al*., suggested that using the P measure may reveal more efficient detection of spatial learning^9^.

The new indices reported here are based upon sufficient trajectory information, and they use the accumulation of real-time deviations to quantitatively describe the dynamic processes of animal learning behavior. The aim of the present study is to explore dynamic evaluation indicators based on behavioral processes of animal learning and memory, and to make up for the lack of summative evaluation that only pays attention to the final results in a limited time. Another aim of the present study is to form a comprehensive evaluation system that combines summative and dynamic evaluation for the MWM.

## Results

### New deviation indices

**Figure 1:**
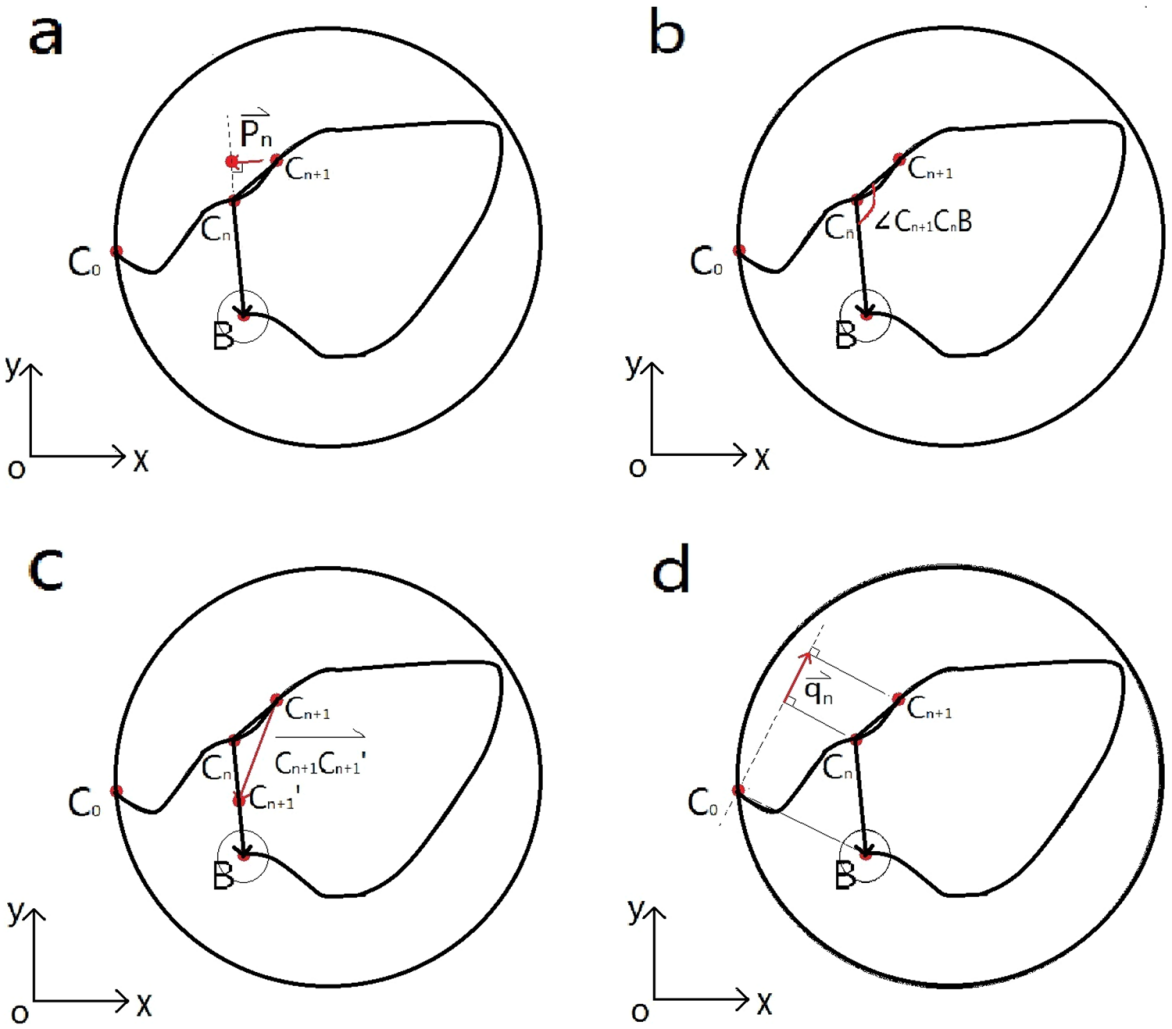
Schematic Diagram of four deviation indices. (**a**) The lateral deviation vectors per unit time $\overrightarrow{{{\rm{p}}}_{{\rm{n}}}}$: it takes C_n+1_ as the starting point and the foot point of C_n+1_ perpendicular to C_n_B as the ending point. (**b**) The offset angle per unit time is ∠C_n+1_C_n_B. (**c**) The correction vectors per unit time $\,\overrightarrow{{{\rm{C}}}_{{\rm{n}}+1}{{\rm{C}}}_{{\rm{n}}+1}^{\prime} }$: it takes C_n+1_ as the starting point and ${{\rm{C}}}_{{\rm{n}}+1}^{\prime}$ as the ending point, representing the addition of correction vectors needed to correct the actual motion trajectory vectors to the same length in the optimal route direction of the corresponding unit time. (**d**) The lateral deviation vectors relative to initial optimal route per unit time $\,\overrightarrow{{{\rm{q}}}_{{\rm{n}}}}$: it takes the foot of perpendicular from C_n_ to the line perpendicular to line C_0_B and passing through point C_0_ as the starting point, and the foot of perpendicular from C_n+1_ to the line perpendicular to line C_0_B and passing through point C_0_ as the ending point.

According to map cognition^12^, experimental subjects can determine their own location and the direction of the platform by establishing a relatively perfect cognitive map, and they can search the platform according to the optimal route. Thus, we proposed an alternative model that measures deviation indices. The new deviation indices refer to the deviation degree of the subject’s trajectory relative to the optimal route in unit time. This degree may consist of several parameters including angle, distance, or some derived index. We proposed four new deviation indices for angle and distance (Fig. 1): the sum of the lateral deviation vectors, the sum of the offset angles, the sum of the correction vectors, and the sum of the lateral deviation vectors relative to the initial optimal route. Different from the summative evaluation indices such as escape latency, the four new measures provide a dynamic evaluation, emphasizing the dynamic changes of the experimental subjects in the processes of searching for the platform. Using the currently available camera tracking systems, coordinate data on the trajectory and swim speed can be recorded to calculate the four new deviation indices outlined in this publication. The learning and memory ability of the experimental subjects can be measured more accurately by comparing the deviation degree of the motion trajectory with the optimal path using our new measures.

As shown in Fig. 1, Point B is the center of the platform, with coordinate (x_B_, y_B_). Point C_0_ is the entry of the experimental subject, with coordinate (x_0_, y_0_). The curve from C_0_ to B is the trajectory of the experimental subject. Point C_n_ is any point recorded on the trajectory, with coordinate (x_n_, y_n_). Point ${{\rm{C}}}_{{\rm{n}}+1}^{\prime}$ is the recording point that is separated from C_n_ in one unit time t(s), with coordinate (x_n+1_, y_n+1_ (n ∈ N). Point ${{\rm{C}}}_{{\rm{n}}+1}^{\prime}$ is the auxiliary point that makes ${{\rm{C}}}_{{\rm{n}}}{{\rm{C}}}_{{\rm{n}}+1}^{\prime}$ have the same length as C_n_C_n+1_. The optimal route would be directly from Point C_n_ to B and from C_0_ to Point B. The direction of $\overrightarrow{{{\rm{C}}}_{{\rm{n}}}{{\rm{C}}}_{{\rm{n}}+1}}$ is considered as the direction of the experimental subject’s movement in time.

#### The sum of the lateral deviation vectors (index 1)

The lateral deviation vector length $|\overrightarrow{{{\rm{p}}}_{{\rm{n}}}}|$ is the component length of the motion trajectory vectors per unit time in the vertical direction of the optimal route. The calculation method can be described by formula (1).

1 $$|\overrightarrow{{{\rm{p}}}_{{\rm{n}}}}|=\{\begin{array}{ll}|\frac{({{\rm{x}}}_{{\rm{n}}+1}-{{\rm{x}}}_{{\rm{n}}})\cdot ({{\rm{y}}}_{{\rm{B}}}-{{\rm{y}}}_{{\rm{n}}})+({{\rm{y}}}_{{\rm{n}}+1}-{{\rm{y}}}_{{\rm{n}}})\cdot ({{\rm{x}}}_{{\rm{n}}}-{{\rm{x}}}_{{\rm{B}}})}{\sqrt{{({{\rm{y}}}_{{\rm{B}}}-{{\rm{y}}}_{{\rm{n}}})}^{2}+{({{\rm{x}}}_{{\rm{n}}}-{{\rm{x}}}_{{\rm{B}}})}^{2}}}| & ({{\rm{y}}}_{{\rm{n}}}\ne {{\rm{y}}}_{{\rm{B}}}\,{\rm{or}}\,{{\rm{x}}}_{{\rm{n}}}\ne {{\rm{x}}}_{{\rm{B}}})\\ 0 & ({{\rm{y}}}_{{\rm{n}}}={{\rm{y}}}_{{\rm{B}}}\,{\rm{and}}\,{{\rm{x}}}_{{\rm{n}}}={{\rm{x}}}_{{\rm{B}}})\end{array}$$

The ${\rm{sum}}={\sum }_{{\rm{n}}=0}^{{{\rm{t}}}_{{\rm{escape}}{\rm{latency}}}\times {\rm{record}}\,{\rm{frequency}}-1}|\overrightarrow{{{\rm{p}}}_{{\rm{n}}}}|$ is the sum of the component lengths of all the motion trajectory vectors in the vertical direction of the real-time optimal route for every unit time. Index 1 accumulates the amount of movement in the direction perpendicular to the optimal route, reflecting the extra distance consumed in the vertical direction of the optimal route by the experimental subject in the process of searching for the platform.

#### The sum of the offset angles (index 2)

The offset angle ∠C_n+1_C_n_B is the deviation angle between the motion trajectory vector and the optimal route in the unit time. The calculation method is listed below as formula (2).

2 $$\cos \,\angle {{\rm{C}}}_{{\rm{n}}+1}{{\rm{C}}}_{{\rm{n}}}{\rm{B}}=\{\begin{array}{c}\frac{({{\rm{x}}}_{{\rm{n}}+1}-{{\rm{x}}}_{{\rm{n}}})\cdot ({{\rm{x}}}_{{\rm{B}}}-{{\rm{x}}}_{{\rm{n}}})+({{\rm{y}}}_{{\rm{n}}+1}-{{\rm{y}}}_{{\rm{n}}})\cdot ({{\rm{y}}}_{{\rm{B}}}-{{\rm{y}}}_{{\rm{n}}})}{\sqrt{{({{\rm{x}}}_{{\rm{n}}+1}-{{\rm{x}}}_{{\rm{n}}})}^{2}+{({{\rm{y}}}_{{\rm{n}}+1}-{{\rm{y}}}_{{\rm{n}}})}^{2}}\times \sqrt{{({{\rm{x}}}_{{\rm{B}}}-{{\rm{x}}}_{{\rm{n}}})}^{2}+{({{\rm{y}}}_{{\rm{B}}}-{{\rm{y}}}_{{\rm{n}}})}^{2}}}\\ (({{\rm{x}}}_{{\rm{n}}+1}\ne {{\rm{x}}}_{{\rm{n}}}\,{\rm{or}}\,{{\rm{y}}}_{{\rm{n}}+1}\ne {{\rm{y}}}_{{\rm{n}}})\,{\rm{and}}\,({{\rm{x}}}_{{\rm{B}}}\ne {{\rm{x}}}_{{\rm{n}}}\,{\rm{or}}\,{{\rm{y}}}_{{\rm{B}}}\ne {{\rm{y}}}_{{\rm{n}}}))\\ 0({{\rm{x}}}_{{\rm{n}}+1}={{\rm{x}}}_{{\rm{n}}},{{\rm{y}}}_{{\rm{n}}+1}={{\rm{y}}}_{{\rm{n}}}\,{\rm{or}}\,{{\rm{x}}}_{{\rm{B}}}={{\rm{x}}}_{{\rm{n}}},{{\rm{y}}}_{{\rm{B}}}={{\rm{y}}}_{{\rm{n}}})\end{array}$$

Then, the offset angle ∠C_n+1_C_n_B is obtained through the inverse trigonometric function (the range of the deviation angle is 0°–180°). The ${\rm{Sum}}={\sum }_{{\rm{n}}=0}^{{{\rm{t}}}_{{\rm{escape}}{\rm{latency}}}\times {\rm{record}}\,{\rm{frequency}}-1}\angle {{\rm{C}}}_{{\rm{n}}+1}{{\rm{C}}}_{{\rm{n}}}{\rm{B}}$ is the sum of the offset angles of all the motion trajectory vectors per unit time. Index 2 accumulates all angle values between the actual trajectories of the experimental subjects and the directions of the real-time optimal route for every unit time. It represents the deviation degree of the motion direction from the optimal route in the process of searching for the platform, the construction of the cognitive map of the experimental subject, and the mastery of the real-time relative position between the target point and themselves. It also can illustrate the consistency between the moving direction and the optimal route direction.

#### The sum of the correction vectors (index 3)

The correction vectors $|\overrightarrow{{{\rm{C}}}_{{\rm{n}}+1}{{\rm{C}}}_{{\rm{n}}+1}^{\prime} }|$ are vectors that need to be added to correct the motion trajectory vectors to the same length vectors in the optimal route direction in unit time. The calculation method is listed below as formula (3).

3 $$\begin{array}{c}|\overrightarrow{{{\rm{C}}}_{{\rm{n}}+1}{{\rm{C}}}_{{\rm{n}}+1}^{\prime} }|=\\ \{\begin{array}{c}2\times \sqrt{{({{\rm{x}}}_{{\rm{n}}+1}-{{\rm{x}}}_{{\rm{n}}})}^{2}+{({{\rm{y}}}_{{\rm{n}}+1}-{{\rm{y}}}_{{\rm{n}}})}^{2}}\times \sqrt{\frac{1-(\frac{({{\rm{x}}}_{{\rm{n}}+1}-{{\rm{x}}}_{{\rm{n}}})\cdot ({{\rm{x}}}_{{\rm{B}}}-{{\rm{x}}}_{{\rm{n}}})+({{\rm{y}}}_{{\rm{n}}+1}-{{\rm{y}}}_{{\rm{n}}})\cdot ({{\rm{y}}}_{{\rm{B}}}-{{\rm{y}}}_{{\rm{n}}})}{\sqrt{{({{\rm{x}}}_{{\rm{n}}+1}-{{\rm{x}}}_{{\rm{n}}})}^{2}+{({{\rm{y}}}_{{\rm{n}}+1}-{{\rm{y}}}_{{\rm{n}}})}^{2}}\times \sqrt{{({{\rm{x}}}_{{\rm{B}}}-{{\rm{x}}}_{{\rm{n}}})}^{2}+{({{\rm{y}}}_{{\rm{B}}}-{{\rm{y}}}_{{\rm{n}}})}^{2}}})}{2}\,}\\ (({{\rm{x}}}_{{\rm{n}}+1}\ne {{\rm{x}}}_{{\rm{n}}}\,{\rm{or}}\,{{\rm{y}}}_{{\rm{n}}+1}\ne {{\rm{y}}}_{{\rm{n}}})\,{\rm{and}}\,({{\rm{x}}}_{{\rm{B}}}\ne {{\rm{x}}}_{{\rm{n}}}\,{\rm{or}}\,{{\rm{y}}}_{{\rm{B}}}\ne {{\rm{y}}}_{{\rm{n}}}))\\ 0({{\rm{x}}}_{{\rm{n}}+1}={{\rm{x}}}_{{\rm{n}}},{{\rm{y}}}_{{\rm{n}}+1}={{\rm{y}}}_{{\rm{n}}}\,{\rm{or}}\,{{\rm{x}}}_{{\rm{B}}}={{\rm{x}}}_{{\rm{n}}},{{\rm{y}}}_{{\rm{B}}}={{\rm{y}}}_{{\rm{n}}})\end{array}\end{array}$$

The ${\rm{sum}}={\sum }_{{\rm{n}}=0}^{{{\rm{t}}}_{{\rm{escape}}{\rm{latency}}}\times {\rm{record}}\,{\rm{frequency}}-1}|\overrightarrow{{{\rm{C}}}_{{\rm{n}}+1}{{\rm{C}}}_{{\rm{n}}+1}^{\prime} }|$ is the sum of all lengths of the correction vectors for every unit time. It reflects the extra distance that the experimental subject needs to take in order to correct its motion trajectory to the optimal route in the process of searching for the platform, the degree to which the cognitive map has been considered, and the mastery of the real-time relative position between the target and itself. The value relates to both the deviation angle and the moving speed. Compared to $|\overrightarrow{{{\rm{p}}}_{{\rm{n}}}}|$, it also calculates the opposite length of the movement trajectory in the optimal route. This more accurately and comprehensively reflects the experimental subject’s knowledge of the position of the platform.

#### The sum of the lateral deviation vectors to the initial optimal route (index 4)

The length of the lateral deviation vectors relative to the initial optimal route $|\overrightarrow{{{\rm{q}}}_{{\rm{n}}}}|$ is the projection length of the motion trajectory vectors per unit time in the vertical direction of the optimal route in the initial unit time. The calculation is shown below as formula (4).

4 $$|\overrightarrow{{{\rm{q}}}_{{\rm{n}}}}|=|\frac{({{\rm{x}}}_{{\rm{n}}+1}-{{\rm{x}}}_{{\rm{n}}})\cdot ({{\rm{y}}}_{{\rm{B}}}-{{\rm{y}}}_{0})+({{\rm{y}}}_{{\rm{n}}+1}-{{\rm{y}}}_{{\rm{n}}})\cdot ({{\rm{x}}}_{0}-{{\rm{x}}}_{{\rm{B}}})}{\sqrt{{({{\rm{y}}}_{{\rm{B}}}-{{\rm{y}}}_{0})}^{2}+{({{\rm{x}}}_{0}-{{\rm{x}}}_{{\rm{B}}})}^{2}}}|$$

The ${\rm{sum}}={\sum }_{{\rm{n}}=0}^{{{\rm{t}}}_{{\rm{escape}}{\rm{latency}}}\times {\rm{record}}\,{\rm{frequency}}-1}|\overrightarrow{{{\rm{q}}}_{{\rm{n}}}}|$ is the sum of the projection lengths of the motion trajectory vectors in every unit time in the vertical direction of the optimal route in the initial unit time. Index 4 accumulates the quantity of motion in the direction perpendicular to the initial optimal route, reflecting the extra distance consumed by the experimental subject in the vertical direction of the optimal route in the process of searching for the platform. Compared to $|\overrightarrow{{{\rm{p}}}_{{\rm{n}}}}|$, it emphasizes the mastery degree of the experimental subjects to the position of the entry point, so it can better reflect the formation of the experimental subjects’ sequence habits.

### Normal distribution in control and vascular dementia model group

First, we measured escape latency in the experimental (model) and control groups to calculate the normal distribution. As shown in Table 1, escape latency in 29 out of 40 (72.5%) training sessions did not demonstrate a normal distribution. However, using the new deviation indices (1, 2, 3 and 4), a reduced percent of trials did not show a normal distribution for each index measured (1–4, 22.5%, 45%, 20%, and 17.5%, respectively; Table 1). Both parametric and non-parametric measures were used to evaluate the behavioral differences measured using escape latency versus using the new deviation indices in 20 training sections. Table 2 summarized the results from the statistical analyses using the t-test for data with a normal distribution and the U test for data that did not have a normal distribution. Using escape latency as a measure, only one training result had a normal distribution. However, 11 results for index 1, 7 results for index 2, 12 results for index 3, and 14 results for index 4 had a normal distribution as measured by the t-test. Our results indicate that the new deviation indices are more consistent in their measures than that of escape latency.

**Table 1:** The *p* values of normal distribution test of five indices.

Training batches	Escape latency		Index 1		Index 2		Index 3		Index 4	
	Model	Control	Model	Control	Model	Control	Model	Control	Model	Control
1	0.000*	0.000*	0.043*	0.200	0.001*	0.028*	0.001*	0.119	0.070	0.200
2	0.000*	0.000*	0.200	0.200	0.181	0.105	0.200	0.200	0.200	0.200
3	0.000*	0.011*	0.200	0.147	0.003*	0.121	0.200	0.121	0.175	0.138
4	0.000*	0.000*	0.200	0.200	0.200	0.200	0.200	0.200	0.189	0.200
5	0.000*	0.015*	0.160	0.000*	0.000*	0.000*	0.108	0.000*	0.036*	0.000*
6	0.000*	0.200	0.200	0.073	0.000*	0.094	0.200	0.106	0.136	0.183
7	0.000*	0.055	0.200	0.007*	0.085	0.009*	0.200	0.007*	0.200	0.006*
8	0.003*	0.200	0.017*	0.200	0.038*	0.200	0.099	0.200	0.173	0.200
9	0.000*	0.033*	0.200	0.200	0.200	0.200	0.200	0.200	0.200	0.200
10	0.000*	0.178	0.200	0.000*	0.047*	0.000*	0.200	0.000*	0.200	0.001*
11	0.002*	0.006*	0.200	0.000*	0.021*	0.009*	0.200	0.003*	0.200	0.000*
12	0.000*	0.004*	0.180	0.200	0.045*	0.035*	0.158	0.140	0.143	0.169
13	0.200	0.003*	0.200	0.000*	0.200	0.000*	0.200	0.000*	0.200	0.000*
14	0.005*	0.200	0.182	0.058	0.200	0.180	0.200	0.141	0.200	0.016*
15	0.000*	0.007*	0.200	0.032*	0.200	0.078	0.200	0.060	0.200	0.105
16	0.004*	0.036*	0.200	0.029*	0.102	0.013*	0.200	0.006*	0.200	0.148
17	0.000*	0.007*	0.200	0.113	0.062	0.130	0.200	0.112	0.200	0.066
18	0.000*	0.096	0.200	0.200	0.012*	0.100	0.022*	0.200	0.190	0.200
19	0.007*	0.127	0.064	0.118	0.200	0.128	0.072	0.140	0.140	0.107
20	0.083	0.098	0.200	0.200	0.200	0.027*	0.200	0.086	0.200	0.200

Note: The normal distribution results of 20 training trials for all indices in model and control group. Using single sample K-S test to test the normality, *p < *0*.*05*, which means that the data of this group in this training didn’t conform to the normal distribution.

**Table 2:** The *p* values of t and u test results of the five indices.

Training batches	Escape latency		Index 1		Index 2		Index 3		Index 4	
	t-test	U test	t-test	U test	t-test	U test	t-test	U test	t-test	U test
1	—	0.347	—	0.261	—	0.673	—	0.465	0.773	—
2	—	0.976	0.267	—	0.601	—	0.419	—	0.558	—
3	—	0.020*	0.002**	—	—	0.003**	0.002**	—	0.001**	—
4	—	0.332	0.147	—	0.586	—	0.157	—	0.106	—
5	—	0.001**	—	0.009**	—	0.011*	—	0.013*	—	0.011*
6	—	0.002**	0.001**	—	—	0.002**	0.001**	—	0.001**	—
7	—	0.004**	—	0.004**	—	0.010*	—	0.005**	—	0.011*
8	—	0.559	—	0.933	—	0.555	0.993	—	0.937	—
9	—	0.348	0.382	—	0.512	—	0.523	—	0.568	—
10	—	0.020*	—	0.015*	—	0.012*	—	0.015*	—	0.020*
11	—	0.005**	—	0.007**	—	0.006**	—	0.013*	—	0.011*
12	—	0.795	0.313	—	—	0.500	0.338	—	0.313	—
13^#^	—	0.166	—	0.011*	—	0.038*	—	0.017*	—	0.009**
14	—	0.178	0.114	—	0.115	—	0.127	—	—	0.152
15^#^	—	0.027*	—	0.087	0.058	—	0.054	—	0.065	—
16	—	0.381	—	0.227	—	0.384	—	0.249	0.168	—
17	—	0.410	0.262	—	0.299	—	0.325	—	0.327	—
18	—	0.474	0.297	—	—	0.216	—	0.399	0.258	—
19	—	0.009**	0.008**	—	0.013*	—	0.008**	—	0.009**	—
20	0.685	—	0.943	—	—	0.448	0.806	—	0.729	—

Note: The table above shows 20 training results. Referring to Table 1, t-test was used if the data of the model and the control group were both in normal distribution, otherwise U test was used. *p < *0*.*05*, **p < *0*.*01*, —missing value because respective test cannot be used, ^#^the result of escape latency is inconsistent with that of new indices in this training.

### Monte Carlo simulation

**Figure 2:**
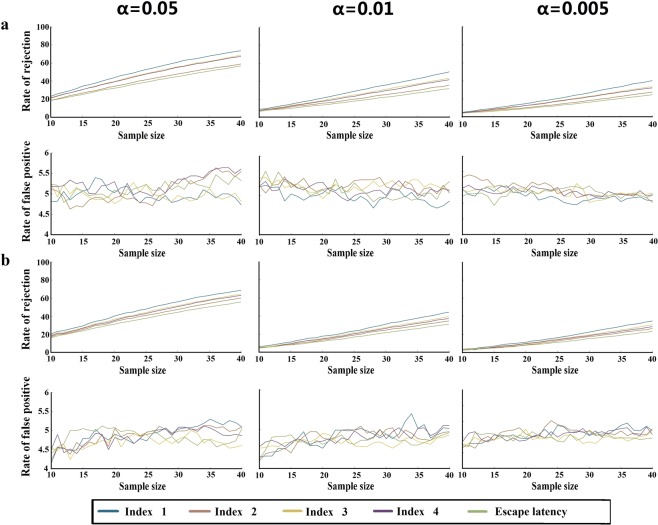
Monte Carlo Simulation. (**a**) T-test. First row of line graphs: rejection rates of the H0 hypothesis between control and model groups varying with sample size at different significance levels α = 0.05, 0.01 and 0.005. Second row of line graphs: false positive rates varying with sample size at different significance levels. (**b**) U test. Third row of line graphs: rejection rates of the H0 hypothesis varying with sample size and significance levels α = 0.05, 0.01 and 0.005. Fourth row of line graphs: false positive rates varying with sample size at different significance levels. The sample size ranges from 10 to 40. For each additional sample, a point was recorded.

The Monte Carlo simulation can simulate a large number of training sessions to examine the sensitivity and specificity of each index^13^. We first collected the experimental data from both groups (obtained by the same researchers using the same experimental subjects and the same equipment in the same environment) to build the database in order to establish a probability model. As shown in Fig. 2, the Monte Carlo simulation was used to perform 10,000 computer simulations with the data obtained from 780 trainings, incorporating the five indices at different significance levels (α = 0.05, 0.01 and 0.005). Using parametric (t-test) and nonparametric tests (U test), we compared the differences between the false positive rates and rejected the null hypothesis in the control and experimental model group. Multiple simulations of different sample sizes were used to compare escape latency measures to measures that used the four indices. Table 2 demonstrated that using new indices that measured the false positive rate of each index was close to the predicted value (*p* > 0.05), and the true positive rates (using the t-test or the U test) were significantly higher than those of escape latency (classic index). Our results demonstrate that the new deviation indices display a higher sensitivity compared to that of the classic escape latency index through using the Monte Carlo simulation test.

### Receiver operating characteristic (ROC) curve

**Figure 3:**
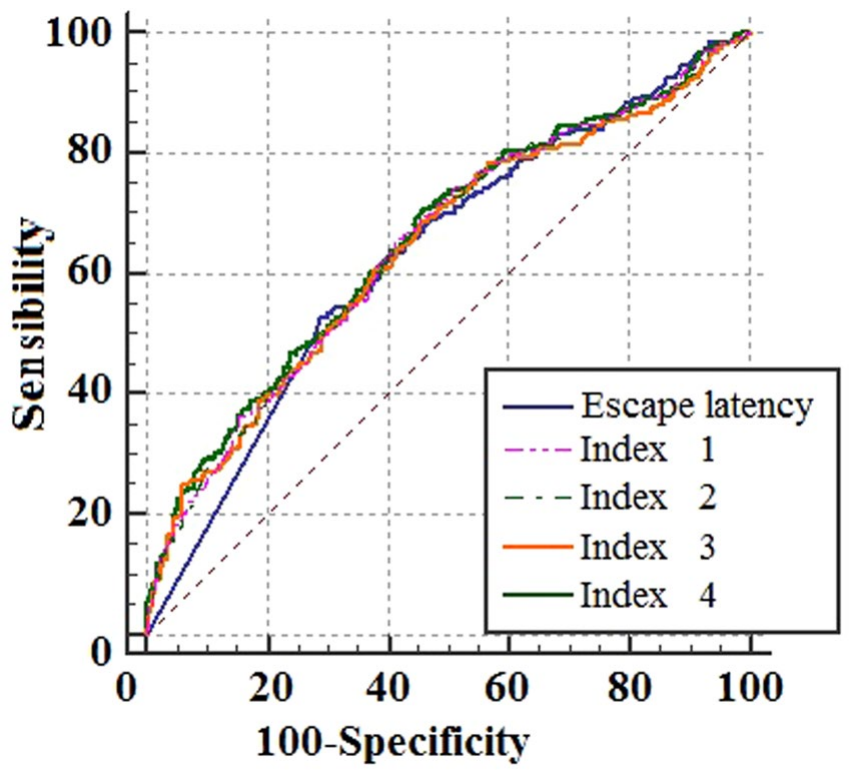
ROC curve of new indices and escape latency. Area under the curve (AUC) of escape latency is 0.632; AUC of index 1 is 0.657; AUC of index 2 is 0.643; AUC of index 3 is 0.649; AUC of index 4 is 0.648.

A ROC curve is a graphical plot that expresses the diagnostic ability of a binary classifier system as its discrimination threshold is varied. It is created by plotting the true positive rate against the false positive rate at various threshold settings^14^. The ROC curve is now widely used to evaluate and compare the diagnostic value of a test^14^. As shown in Fig. 3, the ROC curves were generated based on the 20 training results (19 rats from the dementia model group and 20 rats from the control group). We also compared the differences between the four new indices and escape latency for their sensitivity and specificity (Fig. 3). The ROC curves of the four new indices were clearly distinct from the curve generated using escape latency measures. The area under the curve (AUC) of each new deviation index was larger than that of escape latency, especially when the false positive rate was lower than 20%.

The difference between the new deviation indices and escape latency can be evaluated by AUC. The Z test (examined by using MedCalc software) compares the advantages and disadvantages of each index precisely. Therefore, we compared escape latency with the new indices by using the Z test. Index 1 and index 3 showed significant differences as compared to escape latency. Index 4 showed a close to a significant difference from escape latency measures. There was no significant difference between index 2 and escape latency (Table 3). Taken together, our data suggest that the two ROC curves of the deviation indices, index 1 and 3, have a higher diagnostic value than the classic index (escape latency), while deviation indices 2 and 4 are less predictive. Index 4 showed a close to significant result, and this could indicate that an increased sample size is needed. This will be examined in a future study.

**Table 3:** Comparison of the ROC curves between 4 new indices and escape latency.

	Escape latency -Index 1	Escape latency -Index 2	Escape latency -Index 3	Escape latency -Index 4
Difference between AUC	0.02490	0.01090	0.01720	0.01600
Standard error	0.00830	0.00686	0.00787	0.00818
95% Confidence interval	0.00865–0.04120	−0.00253–0.02440	0.00176–0.03260	−0.00006–0.03200
Z test	3.002	1.591	2.183	1.953
Significant difference	*p* = *0*.*003*	*p* = *0*.*112*	*p* = *0*.*029*	*p* = *0*.*051*

The index values of the subjects should decrease with increasing training times, and a stable index should reflect learning^8^. In a subset of the animals, learning did not increase with increasing the number of training sessions, leading to worse index stability. The statistical data showed that using escape latency measures, 346 out of 624 subjects did not learn with increased training trials. However, using index 1–4 measures in the trials, only 282, 291, 281, and 277, respectively, did not learn. The new index values showed significant differences as compared to escape latency by paired t-tests (*p* < 0.01). Our data demonstrate that the new indices better reflect the trend of continuous improvement of the experimental subjects during the training process compared to the classically used escape latency index. This reveals more stability in measurements.

### Comparison of the value of using the new deviation indices and escape latency measures between control and dementia group

We further compared the differences between the control and the model group by using the new deviation indices and the classic index (escape latency). As shown in Table 4, there were significant differences in the means and standard deviations (*p* < 0.05, Analysis of Variance by ANOVA) using escape latency measures compared to the new index measures between control and experimental groups in the 20 training sections. The new index measures detected differences in the behavior of the experimental model groups compared to the controls, while escape latency measures failed to detect differences. These results indicate that the new index measures can more precisely detect differences in learning and memory.

**Table 4:** Data of new indices and escape latency in model and control group ($\bar{{\rm{x}}}$ ± SD).

Group	Escape latency (s)	Index 1 (cm)	Index 2 (∠)	Index 3 (cm)	Index 4 (cm)
Control	31.6 ± 22.4	535.9 ± 394.1	60278.5 ± 40001.6	953.1 ± 705.0	485.8 ± 336.7
Model	42.4 ± 22.0*	791.9 ± 471.1*	80814.5 ± 41477.2*	1370.1 ± 797.9*	679.8 ± 375.2*

Note: *p < *0*.*05*, comparing the model and the control group by repetitive measure analysis. Data are presented as means ± standard deviations (SD).

We also analyzed the results of each training by using t-test to determine if the data from both groups had a normal distribution. With the exception of the 13^th^ and 15^th^ training results (Table 5), the five indices were consistent with each other. Table 5 showed the average and standard deviation of the 13^th^ and 15^th^ training results of the model and control group. In the 13^th^ training result, the data for escape latency and the four new indices did not have a normal distribution, so the U test was used. In the 15^th^ training result, the t-test showed significance for a normal distribution when using the new index 2, 3, 4 in the model and the control group. In trial 15, using escape latency measures or index 1 measures, a normal distribution was not observed. Therefore, the U test was used.

**Table 5:** Data of new indices and escape latency in the 13^th^ and 15^th^ training ($\bar{{\rm{x}}}$ ± SD).

Training batches	Group	Escape Latency (s)	Index 1 (cm)	Index 2 (∠)	Index 3 (cm)	Index 4 (cm)
13	Control	25.9 ± 22.0	364.3 ± 317.6	43522.1 ± 34531.2	650.7 ± 582.6	343.2 ± 279.0
	Model	34.9 ± 19.9	716.1 ± 419.0*	70977.8 ± 37403.0*	1219.5 ± 715.8*	631.7 ± 343.5*
15	Control	21.9 ± 22.8	425.7 ± 405.8	45809.2 ± 44262.3	736.8 ± 702.9	375.4 ± 345.5
	Model	39.9 ± 23.5^#^	666.7 ± 419.1	78261.9 ± 43869.7	1206.2 ± 733.9	592.5 ± 348.8

Note: *p < *0*.*05*, comparing the model and the control group in the 13^th^ training. ^#^*p* < *0*.*05*, comparing the model and the control group in the 15^th^ training. Data are presented as means ± standard deviations (SD).

**Figure 4:**
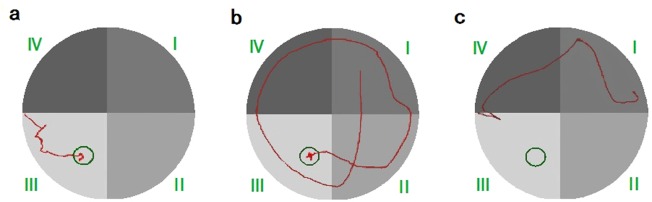
Swimming trajectory of three rats in the model group during the 13^th^ training. (**a**) No. 11; (**b**) No. 15; (**c**) No. 18.

In order to explore the authenticity of these findings, we extracted all of the original data from every rat in both groups (13^th^ and 15^th^ training). In the 13^th^ training (see Supplementary Table S1), rats ^#^9 and ^#^11 demonstrated that escape latency was longer but the deviation index was smaller in the 20 training sessions (see Supplementary Table S2). The training video showed that the motion speed was slower than normal and occasionally, the animals spent more time floating, but the trajectory of the movement almost mapped onto the optimal path (Fig. 4a). Rat ^#^15 from the control group (see Fig. 4b), in contrast, showed that escape latency was shorter but the deviation index value was larger in the 20 training sessions (see Supplementary Table S3). The training video showed that the motion speed was faster than normal, but the movement trajectory showed a large deviation from the optimal path, because the animal used the swim-around-the-edge strategy in most training sessions to find the platform. Rat ^#^18 in the control group found the platform with longer time, but the new deviation index was smaller. The training video showed that the speed was slower than normal, and the animals often spent time floating (Fig. 4c). Escape latency showed that the learning memory ability of the rats was weak, but the new indices showed that the moving trajectory was in line with the optimal path and its learning memory ability was strong. This situation leads to the differences between the results of escape latency and the new indices.

**Figure 5:**
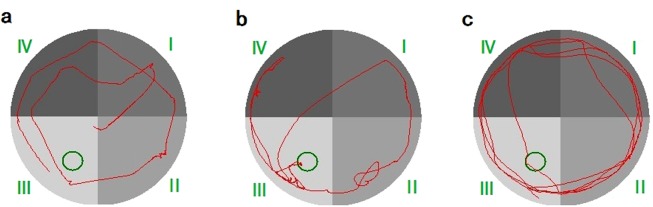
Swimming trajectory of three rats in the model group during the 15^th^ training. (**a**) No. 5; (**b**) No. 8; (**c**) No. 1.

Further, rat ^#^5 and ^#^8 in the model group in the 15^th^ training (see Supplementary Table S4) did not find the platform in the limited time window of escape latency (60 s). However, the new deviation index values for rat ^#^5 and ^#^8 in the model group were smaller compared to that of rat ^#^9 in the model group, who also did not find the platform in 60 s (see Supplementary Table S4). For rat ^#^5 (Fig. 5a) and ^#^8 (Fig. 5b) in the model group that did not find the platform, they were not very active in looking for the platform in all trainings (see Supplementary Table S5). However, rat ^#^9 in the model group (Fig. 5c) spent a longer time to find the platform. It was very active in searching for the platform. Our data further suggest that the new measures reveal better evaluation compared to what escape latency revealed.

## Discussion

### The characteristics and advantages of the new indices

The MWM is widely used to evaluate rodent learning and memory^2^. However, the current evaluation methods are not sensitive enough to detect dynamic changes in learning and memory^9,10^. In order to better evaluate and understand spatial learning and memory in the MWM, we designed and evaluated four new measures, titled deviation index 1–4 in the present study. Index 1, 2, and 3 are more suitable for analyzing the construction of the cognitive maps of the experimental subjects, the mastery degree of the real-time position of the platform, and the development and refinement of search strategies. The successive decrease in value of these three indices with training can illustrate that a cognitive map has been formed by the rodent. Index 4 can be used to better evaluate the list hypothesis theory^15^, which relies on the premise that the subjects do not know whether there is food in the arm of a radial arm maze before entering the arm, and after observing and analyzing the cues outside the arm (micro-selection), they can make an informed decision whether to enter the arm or not. According to the list hypothesis theory, the whole training process is a series of decisions in which the subjects make micro-selections and large selections continuously in front of each arm. The subjects will also continue to make left versus right micro-selections while looking for the platform in the water maze. So, index 4 can be applied to determine the motion strategies of the experimental subjects in training under the same experimental environment (especially the same entry point) and to evaluate the mastery degree of the real-time position between the entry point and the platform.

In the present study, we examined the normal distribution of the data generated from the new deviation indices and escape latency, and evaluated the sensitivity and diagnostic value using the Monte Carlo simulation and ROC curve analyzations. Our results demonstrate that using the new deviation indices revealed higher consistency as compared to using the classic escape latency index. Our data suggest that the new indices might be an alternative method to evaluate the dynamic changes in animal space exploration as there is a deficiency in the simple classifications of the current searching strategies.

### Analysis of the reasons for the differences between escape latency and the new deviation indices in the thirteenth and fifteenth training results

We further analyzed the results of every single training. The results showed that escape latency and deviation index clearly differed in the results of the 13^th^ and 15^th^ training. We found that the significant differences were caused mainly by two situations: (1) The experiment subjects used less time to find the platform, but they demonstrated a greater deviation from the optimal search route. This led to the shorter escape latency and higher deviation index. This might be due to the inability of the subject to construct a cognitive map between itself and the platform position. The decrease in escape latency is mainly due to the fast moving that caused an increased probability of meeting the platform. (2) The experiment subjects took a longer time to find the platform, but they demonstrated decreased deviation from the optimal search route, which led to the longer escape latency but smaller deviation index value. This was due to the fact that the experimental subjects did not recognize the relationship between the platform and the escape^16–18^. We might speculate that the water maze environment may not produce enough stimulation and force the rat to escape, which is contrary to the principle of the MWM^18^. In this situation, we could not evaluate their learning and memory ability through escape latency and the new deviation indices. It could be ruled out by the maximum escape latency and the smaller deviation indices.

Our present study suggests that the results of the new deviation indices in the 13^th^ training are more reasonable. The results of the 15^th^ training should be reconsidered after ruling out subjects who were not active in the training. In the process of training after training, if experimental subjects were not active, then escape latency and the new deviation indices cannot correctly describe their learning and memory ability. We can filter out the experimental subjects by comparing the values of escape latency and the new deviation indices to ensure the accuracy of the analysis results. Due to escape latency and the new deviation indices in the 15^th^ training not showing significant differences, the four new indices not only could be used as a useful supplement to escape latency, but also could be combined with escape latency to eliminate the interference of some passive experimental subjects in the experiment, keeping the experimental evaluation more reasonable.

### Statistical comparison between escape latency and new deviation indices

The data on escape latency mostly did not align with the normal distribution each time because of the assignment operator of the maximum escape latency score (60 s), when applying to all subjects who failed to find the platform. Non-normal distribution will increase type I error rate to some extent^19^, because t-tests pay more attention to the value of the data than does the rank-sum (K–S) test, making the data of non-normal distribution prone to reject the correct hypothesis of H0, and then impossible to guarantee the test for homogeneity of variance and t-test facticity^19^. By contrary, the new deviation indices paid little attention to the training completion of the rats and never assigned the same value to the results of the subjects who had not found the platform, so the proportion of the new deviation indices’ data not conforming to the normal distribution was relatively small, which improved the test efficiency.

Through the Monte Carlo simulation, we found that the true positive rate (rejection rate of H0 hypothesis) of the new indices of any sample size in the t and U test was higher than that of escape latency, indicating that the sensitivity of each new index was higher than that of escape latency. Therefore, using the new deviation indices to evaluate the effect of the dementia model might be more sensitive to distinguish the behavioral differences between the model and the control group. In addition, the Monte Carlo simulation also showed that the false positive rate of both the new indices and escape latency are close to the predicted value.

In the circumstance that escape latency and the new deviation indices results are paradoxical, the new deviation indices may reflect the quality of the movement process to evaluate the learning and memory ability of the experimental subjects, with less inclination to misjudge compared to how escape latency does. Therefore, only using escape latency as an index to measure the learning and memory ability may lead to errors in experimental conclusions.

The ROC curve also distinctly shows the dominant position of the new deviation indices on sensitivity and specificity. Especially when the false positive rate is less than 20%, the AUC of the new deviation indices is clearly higher than that of escape latency, showing higher sensitivity. The phenomenon of escape latency ROC curve being a straight line when the values on the x axis are between 0 and 27.75% is due to the limited time window of escape latency (60 s). The false positive rate on the x axis of the ROC curve is directly crossed from 0% to 27.75% because nearly 30% of escape latency is 60 s, and this section can be observed as a straight line from the ROC curve. It can be concluded that escape latency sometimes may not ensure better specificity.

The new deviation indices focus on the dynamic changes of learning and memory, rather than time. They can finely describe the motion characteristics of the experimental subject during the whole process of searching for the platform, which is helpful to observe their dynamic changes of behavior in the training process. The new deviation indices are helpful to the standardized preservation of the experimental results of the MWM and the more efficient use of the experimental data and the establishment of a database.

In conclusion, the new deviation indices are not only consistent with escape latency, but also have better normal distribution, sensitivity, specificity and authenticity than escape latency alone. The new deviation indices combined with escape latency may more accurately analyze the unique behaviors and the learning and memory strategies of experimental subjects in each training. The combination of the new deviation indices and escape latency makes it possible to establish a comprehensive evaluation system, which may address the dynamic process, learning strategies, and the summative assessment. Meanwhile, a more reasonable and standardized analysis method of animal learning processes will be established with the trajectory data by developing new software.

## Materials and Methods

### The rat vascular dementia model

39 male Sprague-Dawley (SD) rats, weighing 200 ± 20 g, were maintained on a 12-h/12-h light/dark (7:00 am on and 7:00 pm off) cycle in a temperature- and humidity-controlled facility with food and water available ad libitum. Rats, provided by Animal Experimental Center of Qiqihar Medical University, were randomly divided into two groups (model group, n = 19 and control group, n = 20). The housing environment was maintained at 23 ± 1 °C and humidity at 50 ± 10%. The light in the training condition of MWM was 10–20 lux. The water temperature in the tank was 22 ± 1 °C. All the behavioral training of the rats was completed between 7:00 am and 7:00 pm. All procedures were conducted in accordance with standards of laboratory animal care and used policy approved by the Institutional Animal Care and Use Committee of Qiqihar Medical University.

Double-side vascular occlusion (2-VO, common carotid artery permanent ligation) in the rats can cause long-term chronic cerebral hypoperfusion, leading to deterioration of spatial learning memory ability^20^ and accelerated development of dementia^21,22^. In the dementia model group, the bilateral common carotid artery was ligated according to modified 2-VO^23^. The operation was done in a completely aseptic state. The rats were injected intraperitoneally with 0.5% sodium pentobarbital (Beijing Lan Tai Chemical Technology Co. Ltd., China) according to their weight (50 mg/kg). The anesthesia level of the rats was determined by the disappearance of corneal reflex, righting reflex and toe response. The anesthesia process was smooth, and the rats were in a stable breathing condition during the operation. When the appropriate level of surgical anesthesia was reached, the rats were fixed on the operating table in the supine position, disinfecting skin, opening a 1–1.5 cm incision along the side of the neck, blunting dissection subcutaneous tissue and muscle, determining the common carotid sheath and separating the common carotid artery from nerves by a glass needle, ligating the unilateral common carotid artery permanently with surgical sutures, then using gentamicin sulfate (Wuhan Aimin Pharmaceutical Co. Ltd., China) 3–5 drops (0.1 ml/100 g) in the incision to prevent infection. The rats were recovered 20 to 30 min later after surgery. Postoperative analgesia of rats was performed when needed with injected amount of less than 20% of initial volume. After operation, the rats were placed under a warm lamp and put back into cages. In order to ensure the effectiveness of the dementia model, the other side of the common carotid artery of the rat was ligated in the same way three days later. The method for the control group was the same as the model group without ligation of the carotid artery.

All subjects had no symptoms of infection. In any training, there was no significant difference in swimming speed between the two groups (*p* > 0.05), indicating that the injury of the operation did not lead to a significant difference in exercise ability between the two groups of subjects^18^. All rats were used for the MWM test four weeks after surgery.

### Hidden platform training

The rat dementia model in the present study was consistent with the chronic cerebral hypoperfusion response in human aging and dementia^21^, and the impairment of spatial reference and working memory can be evaluated by the MWM test^3^. The MWM device (YiShu Information Technology Co. Ltd., Shanghai, China) used in this experiment consisted of a black stainless-steel round tank (160 cm in diameter, 60 cm in height), transparent plexiglas platform (10 cm in diameter), a surrounding light-blocking curtain, and four marks of different shapes around the tank^18^. The platform was made of transparent material and was submerged 0.5–1 cm below the water surface and located in the center of a certain quadrant (called the target quadrant) selected randomly in four quadrants, making it invisible. The pool was filled with a depth of 40 cm water and replaced regularly. The device was placed in a dimly-lit room, and white dye was not used because of the SD rats’ white fur^24^. The experimenter and other possible clues remained in the same position throughout the experiment, and the room was kept quiet to avoid the stress reaction of rats^25^. In addition, it was necessary to clean up the feces left in the pool regularly to reduce the effect of smell information on rats^26^.

Prior to the commencement of training, all subjects were placed in the water in turn by the experimenter who did not know their grouping situation. It was made sure that they were face up to the maze wall and allowed to swim freely for 120 s to familiarize themselves with the training environment^27,28^. In the course of formal training, they were placed into the pool from four quadrants in sequence, facing the wall; the interval of each training was more than 1 hour, and the training lasted for 5 days^1,18^. The training time was set to 60 s and recorded by an elevated video camera connected to the computer. Swimming paths were recorded from which we could figure out the value of the new deviation indices and escape latency. If the experimental subjects failed to find the platform in 60 s during the first training, they would be guided to the platform and would stay there for 10 s. Escape latency of the subjects who did not find the platform would be recorded as 60 s. All rats were dried with a towel during training intervals and placed in a warm and dry cage^24^.

### Experimental data and statistical analysis

The experimental data included the 20 training escape latencies and the four new deviation indices: index 1, 2, 3, and 4. All behavioral data was analyzed using SPSS24.0 version statistical software package. Independent t-test and repeated measure variance analysis (ANOVA) were used to analyze the statistical differences between the two groups^29^. The probability level is *P* < 0.05. Using SPSS24.0, the normal distribution was conducted. The distribution of normality was tested by single sample K-S test. Using MATLAB 2017A, the Monte Carlo simulation was employed to calculate the sensitivity and false positive rate of the classic escape latency and the four new deviation indices, and the test level was set with α = 0.05, 0.01 and 0.005. MedCalc software was applied to draw ROC curves to determine the sensitivity and specificity of each index (the new deviation indices and escape latency).

## Supplementary information

Supplementary file

## Data Availability

The datasets generated during the current study are available from the corresponding author on reasonable request.

## References

[1] Morris R (1984). Developments of a water-maze procedure for studying spatial learning in the rat. J Neurosci Methods.

[2] D’Hooge R, De Deyn PP (2001). Applications of the Morris water maze in the study of learning and memory. Brain Res Brain Res Rev.

[3] Nunez J (2008). Morris water maze experiment. J Vis Exp.

[4] Cazakoff BN, Johnson KJ, Howland JG (2010). Converging effects of acute stress on spatial and recognition memory in rodents: a review of recent behavioural and pharmacological findings. Prog Neuropsychopharmacol Biol Psychiatry.

[5] Dvorkin A, Benjamini Y, Golani I (2008). Mouse cognition-related behavior in the open-field: emergence of places of attraction. PloS Comput Biol.

[6] Schenk F, Morris RG (1985). Dissociation between components of spatial memory in rats after recovery from the effects of retrohippocampal lesions. Exp Brain Res.

[7] Graziano A, Petrosini L, Bartoletti A (2003). Automatic recognition of explorative strategies in the Morris water maze. J Neurosci Methods.

[8] Paul CM, Magda G, Abel S (2009). Spatial memory: theoretical basis and comparative review on experimental methods in rodents. Behav Brain Res.

[9] Maei HR, Kirill Z, Teixeira CM, Frankland PW (2009). What is the most sensitive measure of water maze probe test performance?. Front Integr Neurosci.

[10] Lindner MD (1997). Reliability, distribution, and validity of age-related cognitive deficits in the Morris water maze. Neurobiol Learn Mem.

[11] Dan V, Golani I, Mitra PP (2007). Analysis of the trajectory of drosophila melanogaster in a circular open field arena. Plos One.

[12] Tolman EC (1948). Cognitive maps in rats and men. Psychol Rev.

[13] Raeside DE (1976). Monte Carlo principles and applications. Phys Med Biol.

[14] Hanley JA, Mcneil BJ (1982). The meaning and use of the area under a receiver operating characteristic (roc) curve. Radiology.

[15] Brown MF, Rish PA, Vonculin JE, Edberg JA (1993). Spatial guidance of choice behavior in the radial-arm maze. J Exp Psychol Anim Behav Process.

[16] Saucier D, Hargreaves EL, Boon F, Vanderwolf CH, Cain DP (1996). Detailed behavioral analysis of water maze acquisition under systemic NMDA or muscarinic antagonism: nonspatial pretraining eliminates spatial learning deficits. Behav Neurosci.

[17] Day LB, Weisand M, Sutherland RJ, Schallert T (1999). The hippocampus is not necessary for a place response but may be necessary for pliancy. Behav Neurosci.

[18] Vorhees CV, Williams MT (2006). Morris water maze: procedures for assessing spatial and related forms of learning and memory. Nat Prot.

[19] Sawilowsky SS, Hillman SB (1992). Power of the independent samples t test under a prevalent psychometric measure distribution. J Consult Clin Psychol.

[20] Farkas E, Luiten PG, Bari F (2007). Permanent, bilateral common carotid artery occlusion in the rat: a model for chronic cerebral hypoperfusion-related neurodegenerative diseases. Brain Res Rev.

[21] de la Torre JC (2000). Impaired cerebromicrovascular perfusion. Summary of evidence in support of its causality in Alzheimer’s disease. Ann N Y Acad Sci.

[22] Farkas E, Luiten PG (2001). Cerebral microvascular pathology in aging and Alzheimer’s disease. Prog Neurobiol.

[23] Mei J, Zhang Y, Zhang B (2000). Improvement and application of multi-infarct dementia model in rats. Zhongguo Zhong Xi Yi Jie He Za Zhi.

[24] Deng-Bryant Y, Leung LY, Caudle K, Tortella F, Shear D (2016). Cognitive Evaluation Using Morris Water Maze in Neurotrauma. Methods Mol Biol.

[25] Fagan WF (2013). Spatial memory and animal movement. Ecol Lett.

[26] Azzubaidi MS, Saxena AK, Talib NA, Ahmed QU, Dogarai BB (2012). Protective effect of treatment with black cumin oil on spatial cognitive functions of rats that suffered global cerebrovascular hypoperfusion. Acta Neurobiol Exp.

[27] Kasza A (2017). Studies for improving a rat model of Alzheimer’s disease: icv administration of well-characterized β-amyloid 1–42 oligomers induce dysfunction in spatial memory. Molecules.

[28] Saucier D, Cain DP (1995). Spatial learning without NMDA receptor-dependent long-term potentiation. Nature.

[29] Park E, Cho M, Ki CS (2009). Correct use of repeated measures analysis of variance. Korean J Lab Med.

