# Supplementary material for: Dynamic Evaluation Indices in Spatial Learning and Memory of Rat Vascular Dementia in the Morris Water Maze
Source: Sci Rep. 2019 May 10;9:7224. doi: 10.1038/s41598-019-43738-x (PMC6510771; doi:10.1038/s41598-019-43738-x)
Supplement: Supplementary file 1 — Supplementary file [file 41598_2019_43738_MOESM1_ESM.docx]

**Dynamic evaluation indices in spatial learning and memory of rat vascular dementia in the Morris water maze**

# Ze Yuan^1^, Hongying Zhou^1^, Ni Zhou^1^, Dong Dong^1^, Yuyang Chu^2^, Junxian Shen^3^, Yunfeng Han^4^, Xiang-Ping Chu^1, 5*^, Kunjie Zhu^6*^

**Supplementary Table S1. The analysis of 4 new indices and escape latency in the 13^th^ training.**

| **Group** | **Number** | **Index value** | | | | |
| --- | --- | --- | --- | --- | --- | --- |
|  |  | **Escape latency**  **(s)** | **Index 1**  **(cm)** | **Index 2**  **(**$\angle)$ | **Index 3**  **(cm)** | **Index 4**  **(cm)** |
| **Control** | 11 | 46.04 | 133.4263 | 18562.07 | 218.114 | 141.8558 |
| **Control** | 18 | 60 | 219.7732 | 29076.07 | 454.910 | 219.4552 |
| **Control** | 9 | 46.76 | 440.0501 | 66796.77 | 801.342 | 435.0043 |
| **Model** | 15 | 18.76 | 659.508 | 44463.19 | 1032.027 | 530.1642 |

Note: lists the values of four rats with the most significant difference between the escape latency and the new index value.

|  | **Index value** | | | | |
| --- | --- | --- | --- | --- | --- |
| **Training batches** | **Escape latency**  **(s)** | **Index 1**  **(cm)** | **Index 2**  **(**$\angle)$ | **Index 3**  **(cm)** | **Index 4**  **(cm)** |
| 1 | 60.0 | 1087.0 | 111297.8 | 1850.7 | 915.6 |
| 2 | 60.0 | 893.8 | 104888.9 | 1507.9 | 714.0 |
| 3 | 60.0 | 713.0 | 104030.0 | 1391.4 | 714.7 |
| 4 | 21.7 | 412.0 | 41340.3 | 663.2 | 360.1 |
| 5 | 13.8 | 259.7 | 31937.7 | 439.2 | 298.3 |
| 6 | 60.0 | 814.9 | 107634.1 | 1531.4 | 679.3 |
| 7 | 59.0 | 932.8 | 110019.0 | 1737.3 | 868.6 |
| 8 | 50.5 | 676.2 | 84685.5 | 1236.8 | 622.9 |
| 9 | 60.0 | 667.9 | 93943.9 | 1272.2 | 623.7 |
| 10 | 25.0 | 288.0 | 31609.9 | 474.3 | 267.6 |
| 11 | 1.0 | 60.1 | 10723.3 | 112.7 | 61.7 |
| 12 | 60.0 | 432.5 | 70739.5 | 843.7 | 496.3 |
| 13 | 46.0 | 133.4 | 18562.1 | 218.1 | 141.9 |
| 14 | 27.8 | 284.2 | 34669.3 | 438.1 | 222.4 |
| 15 | 29.2 | 389.0 | 60889.1 | 774.5 | 396.8 |
| 16 | 18.8 | 205.7 | 31924.2 | 370.1 | 217.6 |
| 17 | 34.0 | 221.2 | 33774.9 | 388.6 | 245.5 |
| 18 | 60.0 | 761.3 | 104544.8 | 1461.8 | 669.5 |
| 19 | 1.4 | 50.4 | 8419.1 | 88.7 | 50.6 |
| 20 | 60.0 | 620.4 | 92370.1 | 1210.2 | 670.4 |

**Supplementary Table S2. All training results of rat ^#^11 in the control group.**

**Supplementary Table S3. All training results of rat ^#^15 in the model group.**

|  | **Index value** | | | | |
| --- | --- | --- | --- | --- | --- |
| **Training batches** | **Escape latency**  **(s)** | **Index 1**  **(cm)** | **Index 2**  **(**$\angle)$ | **Index 3**  **(cm)** | **Index 4**  **(cm)** |
| 1 | 60.0 | 1570.7 | 120378.0 | 2541.4 | 1171.7 |
| 2 | 60.0 | 1615.0 | 130690.3 | 2653.4 | 1337.8 |
| 3 | 60.0 | 1748.9 | 132628.6 | 2824.0 | 1364.4 |
| 4 | 60.0 | 2187.0 | 127139.7 | 3494.4 | 1630.4 |
| 5 | 60.0 | 1957.4 | 126682.2 | 3158.9 | 1463.5 |
| 6 | 60.0 | 1668.2 | 129259.8 | 2694.0 | 1273.4 |
| 7 | 60.0 | 1240.5 | 118162.9 | 2153.9 | 1019.4 |
| 8 | 9.1 | 305.3 | 25235.6 | 511.5 | 300.0 |
| 9 | 32.0 | 855.9 | 70110.6 | 1370.2 | 721.0 |
| 10 | 17.4 | 420.2 | 39701.6 | 684.8 | 307.9 |
| 11 | 38.1 | 1134.9 | 99123.7 | 1922.8 | 952.5 |
| 12 | 60.0 | 1334.0 | 124930.7 | 2352.7 | 1165.0 |
| 13 | 18.8 | 659.5 | 44463.2 | 1032.0 | 530.2 |
| 14 | 39.9 | 925.0 | 87449.1 | 1559.3 | 743.7 |
| 15 | 22.2 | 550.5 | 54010.6 | 958.4 | 484.7 |
| 16 | 21.6 | 512.5 | 47486.4 | 866.5 | 522.8 |
| 17 | 19.2 | 486.7 | 41538.8 | 786.8 | 420.2 |
| 18 | 36.0 | 810.7 | 82700.3 | 1466.6 | 652.2 |
| 19 | 10.0 | 316.5 | 27327.5 | 548.1 | 292.1 |
| 20 | 25.1 | 538.4 | 53821.4 | 923.8 | 583.6 |

**Supplementary Table S4. New indices and escape latency of three rats in the 15^th^ training.**

| **Group** | **number** | **Index value** | | | | |
| --- | --- | --- | --- | --- | --- | --- |
|  |  | **Escape latency**  **(s)** | **Index 1**  **(cm)** | **Index 2**  **(**$\angle$**)** | **Index 3**  **(cm)** | **Index 4**  **(cm)** |
| **Model** | 5 | 60 | 602.1361 | 84777.96 | 1105.278 | 538.4961 |
| **Model** | 8 | 60 | 712.6552 | 86505.2 | 1275.566 | 580.6603 |
| **Model** | 9 | 60 | 1477.442 | 135258.2 | 2502.612 | 1193.951 |

Note: lists the values of tree rats with the most significant difference between escape latency and new indexes value.

**Supplementary Table S5. All training results of rat ^#^5 in the model group.**

|  | **Index value** | | | | |
| --- | --- | --- | --- | --- | --- |
| **Training batches** | **Escape latency**  **(s)** | **Index 1**  **(cm)** | **Index 2**  **(**$\boldsymbol{\angle}$**)** | **Index 3**  **(cm)** | **Index 4**  **(cm)** |
| 1 | 60.0 | 1346.0 | 112942.9 | 2216.9 | 1098.9 |
| 2 | 59.0 | 1027.0 | 96063.7 | 1754.2 | 790.0 |
| 3 | 43.0 | 872.9 | 75309.7 | 1448.9 | 788.0 |
| 4 | 45.9 | 897.0 | 91130.3 | 1573.1 | 901.1 |
| 5 | 60.0 | 957.1 | 106584.9 | 1659.0 | 791.6 |
| 6 | 60.0 | 468.8 | 74837.9 | 949.7 | 512.9 |
| 7 | 38.9 | 417.5 | 55692.1 | 756.2 | 429.2 |
| 8 | 34.3 | 540.2 | 66659.2 | 939.9 | 517.4 |
| 9 | 60.0 | 519.6 | 78791.8 | 988.2 | 483.5 |
| 10 | 60.0 | 921.3 | 99033.1 | 1582.6 | 651.0 |
| 11 | 60.0 | 1022.6 | 110269.9 | 1912.1 | 888.8 |
| 12 | 60.0 | 425.1 | 58293.0 | 703.2 | 400.8 |
| 13 | 41.5 | 374.9 | 57877.7 | 707.4 | 384.7 |
| 14 | 60.0 | 585.1 | 81116.9 | 1041.8 | 391.3 |
| 15 | 60.0 | 602.1 | 84777.9 | 1105.3 | 538.5 |
| 16 | 32.8 | 323.1 | 42068.5 | 515.8 | 302.7 |
| 17 | 60.0 | 461.9 | 71957.1 | 869.9 | 474.1 |
| 18 | 60.0 | 631.1 | 87688.0 | 1107.1 | 495.5 |
| 19 | 60.0 | 657.0 | 92275.0 | 1273.0 | 559.7 |
| 20 | 60.0 | 459.4 | 80355.6 | 923.0 | 515.9 |
